# Electroencephalographic abnormalities in children with type 1 diabetes mellitus: a prospective study

**DOI:** 10.55730/1300-0144.5749

**Published:** 2023-10-12

**Authors:** Ruken YILDIRIM, Ceren GÜNBEY

**Affiliations:** 1 Department of Pediatric Endocrinology, Diyarbakır Children’s Hospital, Diyarbakır, Turkiye; 2 Department of Pediatric Neurology, Diyarbakır Children’s Hospital, Diyarbakır, Turkiye

**Keywords:** Type 1 diabetes mellitus, glutamic acid decarboxylase 65 autoantibodies, epilepsy

## Abstract

**Background/aim:**

The aim herein was to investigate epileptiform discharges on electroencephalogram (EEG), their correlation with glutamic acid decarboxylase 65 autoantibody (GAD-ab) in newly diagnosed pediatric type 1 diabetes mellitus (T1DM) patients and interpret their medium-term utility in predicting epilepsy.

**Materials and methods:**

Children presenting with T1DM between July 2018 and December 2019 were included in this prospective longitudinal study. Patients with a history of head injury, chronic illness, neurological disorder, seizure, autism, or encephalopathy were excluded. EEGs were obtained within the first 7 days of diagnosis and later reviewed by a pediatric neurologist. All of the children were clinically followed-up in pediatric endocrinology and neurology clinics for 2 years after their diagnosis.

**Results:**

A total of 105 children (46 male, 43.8%) were included. The mean age at the time of diagnosis was 9.6 ± 4.1 years (range: 11 months–17.5 years). At the time of admission, 24 (22.9%), 29 (27.6%), and 52 (49.5%) patients had hyperglycemia, ketosis, and diabetic ketoacidosis, respectively. GAD-ab was positive in 55 children (52.4%). No background or sleep architecture abnormalities or focal slowing were present on the EEGs. Of the patients, 3 (2.9%) had focal epileptiform discharges. The mean GAD-ab levels of the remaining 102 patients were 7.48 ± 11.97 U/mL (range: 0.01–50.54) (p = 0.2). All 3 children with EEG abnormality had higher levels of GAD-ab (3.59 U/mL, 31.3 U/mL, and 7.09 U/mL, respectively). None of the patients developed epilepsy during the follow-up, although 1 patient experienced Guillain–Barré syndrome (GBS).

**Conclusion:**

The prevalence of epileptiform discharges in the patients was similar to those of previous studies, in which healthy children were also included. No relationship was found between the epileptiform discharges and GAD-ab, and none of the patients manifested seizures during the first 2 years of follow-up of T1DM. These data support the findings of previous studies reporting that T1DM patients with confirmed electroencephalographic abnormalities do not have an increased risk of epilepsy. On the other hand, GBS might be considered as another autoimmune disease that may be associated with T1DM in children.

## 1. Introduction

Metabolic disorders, especially hyperglycemia and hypoglycemia may cause impairment in neurologic functions, decrease the seizure threshold, and thereby provoke seizures. Children with type 1 diabetes mellitus (T1DM) are at an increased risk of developing epilepsy [1,2]. Recurrent unprovoked seizures have been reported in 2 of the 5 major studies conducted exclusively on pediatric patients [1–5]. The reported data on the frequency of electroencephalogram (EEG) abnormalities in T1DM are controversial and suggest an increased rate of epileptiform discharges of up to 11%, with no increased epilepsy prevalence [5–8].

T1DM is a T-cell–mediated autoimmune disease characterized by insulin deficiency due to the loss of insulin-producing pancreatic cells, with a complex pathogenesis involving environmental and multigenetic factors [9,10]. Autoantibodies against glutamic acid decarboxylase (GAD) are one of the major markers of T1DM and play a pathogenic role in the development of the disease. GAD catalyzes the conversion of glutamic acid into inhibitory neurotransmitter gamma-aminobutyric acid. Therefore, GAD 65 autoantibody (GAD-ab) are associated with neurological disorders such as stiff-person syndrome, limbic encephalitis, and epilepsy. On the other hand, GAD-ab can be positive in the normal population [11–13]. Studies have shown GAD-ab seropositivity at increased or normal titers in T1DM patients with epilepsy [4,14,15].

The goal of this study was to determine the utility of EEG investigation in T1DM by prospectively documenting the rate of epileptiform discharges and their correlation with GAD-ab levels and epilepsy in a cohort of newly diagnosed pediatric patients with T1DM.

## 2. Materials and methods

### 2.1. Study population

Children newly diagnosed with T1DM who were hospitalized in the Pediatric Endocrinology Department of Diyarbakır Children’s Hospital, between July 2018 and December 2019, were included in this study. The diagnosis was made according to the criteria defined by the American Diabetes Association [16]. Exclusion criteria included a history of head injury, chronic illness, neurological disorder, febrile/afebrile seizure, and autism. Patients who had encephalopathy at the time of T1DM diagnoses were also excluded. Age, sex, biochemical parameters, T1DM-associated autoantibody status, and main clinical manifestations at the time of diagnosis (hyperglycemia, ketosis, ketoacidosis) were recorded.

### 2.2. Laboratory and EEG analyses

Three T1DM–associated autoantibodies were measured [glutamic acid decarboxylase 65 autoantibodies (GAD-ab), islet cell autoantibodies (ICA), and insulin autoantibodies (IAA)] in all of the children. In addition, thyroid peroxidase antibodies (TPO-ab), thyroglobulin antibodies (Tg-ab), and tissue transglutaminase antibodies (tTGA-ab) tests were performed. Serum autoantibody levels were measured using the chemiluminescence method with a Snibe Maglumi 2000 system (Shenzhen New Industries Biomedical Engineering Co., Ltd. (Briefed as Snibe Co., Ltd.,), Shenzhen, China). Normal ranges for T1DM-associated autoantibodies were as follows: GAD-ab (0–1 U/mL), IAA (0–7 U/mL), ICA (negative: <1 U/mL, borderline: 1–2 U/mL, positive: >2 U/mL).

A 30-min EEG was performed on the patients while they were in the euglycemic state, within the first week of diagnosis. Standard digital EEG was acquired with 21 electrodes, 10–20 electrode placement, and activation procedures of hyperventilation and photic stimulation, including natural sleep whenever possible. EEGs were interpreted by a pediatric neurologist blinded to the clinical status of the children. EEG abnormalities were classified as background abnormality or epileptiform activity. Focal spikes, sharp waves, and generalized spikes and waves were defined as epileptiform discharges.

Definitions of seizure and epilepsy were obtained from the practical clinical definitions of the task force commissioned by the International League Against Epilepsy (ILAE) as: A) an epileptic seizure is a transient occurrence of signs and/or symptoms due to abnormal excessive or synchronous neuronal activity in the brain; B) epilepsy is 1) at least 2 unprovoked (or reflex) seizures occurring >24 h apart; 2) one unprovoked (or reflex) seizure and a probability of further seizures similar to the general recurrence risk (at least 60%) after 2 unprovoked seizures [17]. The study was approved by the Health Sciences University Diyarbakır Gazi Yaşargil Training and Research Hospital Ethical Committee (approval number: 2018-122). Patients were included in the study after obtaining a consent form signed by their parents.

### 2.3. Statistical analysis

Statistical analyses were performed using IBM SPSS Statistics for Windows 22.0 (IBM Corp., Armonk, NY, USA). Descriptive analyses were performed and the GAD-ab levels in patients with and without epileptiform discharges were compared using the Mann–Whitney U test. p < 0.05 was considered statistically significant.

## 3. Results

A total of 105 children (46 males, 59 females) who had been newly diagnosed with T1DM were evaluated (Table 1). The mean age at diagnosis was 9.6 ± 4.1 years (11 months–17.5 years). Of the patients, 24 (22.9%) had hyperglycemia, 29 (27.6%) had ketosis, and 52 (49.5%) had diabetic ketoacidosis at the time of admission; 87 of the 105 (83%) patients had hyperglycemia-associated symptoms. None of the patients experienced any seizures.

At least 1 of the T1DM-associated autoantibodies was positive in 87 (82.9%) patients, most frequently GAD-ab (n = 55, 52.4%), followed by ICA (n = 44, 41.9%) and IAA (n = 12, 11.4%). TPO-ab and tTGA-ab were positive in 3 (2.9%) and 7 (6.7%) patients, respectively. The main findings of the patients are summarized in Table 1.

The median duration between the T1DM diagnosis and the EEG recording was 4 days (3–7 days); 47 patients had both sleep and awake recordings. Awake and sleep EEG recordings were obtained for 34 patients and 24 patients, respectively. No background or sleep architecture abnormalities or focal slowing were recorded; 3 patients (2.9%) had focal epileptiform discharges in the left centrotemporal, right parietal, and left frontal areas, respectively (Table 2). All 3 of these children had high levels of GAD-ab (3.59 U/mL, 31.3 U/mL, and 7.09 U/mL, respectively). None of the patients developed epilepsy or had seizures during their 2-year follow-up. Only 1 patient experienced a neurological disorder, Guillain–Barré syndrome (GBS), with no seizures. A follow-up EEG was available for only 1 of the 3 patients, which revealed no epileptiform discharges.

## 4. Discussion

In the present study evaluating the rate of epileptiform discharges on the EEGs of newly diagnosed T1DM patients, no relationship was found between epileptiform discharges and GAD-ab. Moreover, none of the patients experienced epilepsy during their 2-year follow-up.

The incidence of epileptiform activity in young asymptomatic patients is low and EEG abnormalities do not predict the long-term risk of seizures in healthy individuals with no seizures, no relevant clinical history, or neurological impairment. In fact, the yield of EEG is so low that many countries no longer perform EEG for this purpose, as shown by the results of civil air crew candidates and military aviation screenings [18–20]. For the pediatric age group, series consisting of healthy infants, children, and adolescents found the prevalence of epileptiform discharges as 0.76%–2.9%. None of the children included in these series developed seizures; therefore, EEG had no predictive value without a relevant clinical history [21–23]. The frequency of focal epileptiform discharges detected in 2.9% of the patients with T1DM in the current study was close to the upper limit of reported rates in healthy populations. On the other hand, some studies have reported a high prevalence of epileptic activity on the EEGs of children with T1DM compared to healthy children [6,7]. Several factors such as a young age, severe hypoglycemia, and diabetic ketoacidosis (DKA) were considered to influence epileptiform abnormalities. While no predictive value of these discharges on a clinical diagnosis, i.e., epilepsy, has been reported, the prevalence of epilepsy was found to be increased in adults and children in large cohort studies and a meta-analysis of T1DM [24–26]. Genetic predispositions, the effects of hypoglycemia/hyperglycemia, and vascular damage in the brain may have caused these 2 concomitant disorders [27]. In addition, the immune system has an important role in both T1DM and epilepsy. T1DM is known to be caused by altered immunity, and recent studies have shown the involvement of inflammatory cascades in epileptogenesis and exacerbation of seizures [28]. Furthermore, GAD-Ab, one of the markers of autoimmunity in T1DM, has also been shown in some patients with epilepsy. However, the pathogenicity of GAD-ab remains controversial [29]. In the study of De Sausa et al., 31,601 patients under 20 years of age, who were diagnosed with T1DM, were evaluated. Similar to the general population, the incidence of epilepsy was found to be 0.44% and no correlation between epilepsy and GAD-ab levels, DKA, or severe hypoglycemia was found. The only factor found to be correlated with epilepsy was hypoglycemia with coma. [4]. EEGs were not analyzed in their study. No increased incidence of seizures/epilepsy was found during the 2-year follow-up in the current study. Herein, the incidence of epileptiform discharges and their relationship with serum GAD-ab levels were found to be consistent with the aforementioned recent study [4].

The association of T1DM and other systemic autoimmune disorders, such as autoimmune thyroiditis, celiac disease, and collagen vascular diseases, has been reported previously [30,31]. In the current series, 1 patient who had both epileptiform discharges on the EEG and GAD-ab positivity in the serum experienced GBS within the 1-year follow-up. There are few studies that have reported the association of diabetes with GBS in adult patients; however, to the best of our knowledge, the patient in the present study is the first reported pediatric patient [32]. This may emphasize that GBS might be considered as another autoimmune disorder that may be associated with T1DM in children.

There were limitations in this study. Statistical power analysis was not performed. A group of the patients did not have sleep records. No spectral analysis was performed on the EEGs. Patients were evaluated with only 1 EEG and had no consecutive EEGs. Furthermore, the follow-up duration was relatively short. Despite these limitations, the prospective nature, the number of patients enrolled, and the timing of the EEGs were its main strengths. EEGs were recorded soon after the diagnosis while the patients were euglycemic, in order to minimize the impact of metabolic abnormalities on the EEG. Moreover, children with chronic or neurologic disorders as well as encephalopathy at the time of diagnosis were excluded, resulting in a relatively homogeneous patient group.

In conclusion, no relationship was found between epileptiform discharges and GAD-ab, and none of the patients experienced epilepsy in the 2-year follow-up in this prospective study. The findings support that children with documented EEG abnormalities do not have an increased risk of epilepsy. Longer follow-up and cognitive assessment would be valuable in this patient group.

## Figures and Tables

**Table 1:** Main characteristics of the 105 patients at the time of diagnosis.

Sex	
Male¶	46 (43.8%)
Female¶	59 (56.2%)

Age*	9.6 ± 4.1 years (11 months–17.5 years).

Hemoglobin A1c %*	12.44 ± 2.29

Diabetic ketoacidosis¶	52 (49.5%)

Ketosis¶	29 (27.6%)

Hyperglycemia¶	24 (22.9%)

GAD-ab°*	7.67 ± 12.03 U/mL

* mean ± standard deviation,

¶ number, percentage,

° glutamic acid decarboxylase 65 autoantibody; normal: (0–1) U/mL.

**Table 2:** Main characteristics of the 3 patients with EEG abnormalities.

	Age/sex	GAD-ab (0–1) U/mL	IAA (0–7) U/mL	ICA	HbA1c %	DKA/ketosis	EEG awake/sleep
Patient 1	9.7 years/F	3.59	3.49	(−)	9.2	−/+	+/+, Left frontal discharges
Patient 2	6.8 years/F	31.3	13.97	(+)	8.9	−/+	−/+, Left centrotemporal discharges
Patient 3	11 years/M	7.09	11.3	(+)	12.3	+/−	+/−, Right parietal discharges

F: female; M: male; IAA: insulin autoantibody; ICA: islet cell autoantibody, HbA1c: hemoglobin A1c, EEG: electroencephalogram, DKA: diabetic ketoacidosis.
